# Parkinson’s disease deficits in time perception to auditory as well as visual stimuli – A large online study

**DOI:** 10.3389/fnins.2022.995438

**Published:** 2022-10-20

**Authors:** Zi H. Su, Salil Patel, Oliver Bredemeyer, James J. FitzGerald, Chrystalina A. Antoniades

**Affiliations:** ^1^Medical Sciences Division, NeuroMetrology Lab, Nuffield Department of Clinical Neurosciences, University of Oxford, Oxford, United Kingdom; ^2^Nuffield Department of Surgical Sciences, University of Oxford, Oxford, United Kingdom; ^3^Oxford Functional Neurosurgery, John Radcliffe Hospital, Oxford, United Kingdom

**Keywords:** time perception, Parkinson’s disease, visual stimuli, auditory stimuli, online testing and experimentation

## Abstract

Cognitive deficits are common in Parkinson’s disease (PD) and range from mild cognitive impairment to dementia, often dramatically reducing quality of life. Physiological models have shown that attention and memory are predicated on the brain’s ability to process time. Perception has been shown to be increased or decreased by activation or deactivation of dopaminergic neurons respectively. Here we investigate differences in time perception between patients with PD and healthy controls. We have measured differences in sub-second- and second-time intervals. Sensitivity and error in perception as well as the response times are calculated. Additionally, we investigated intra-individual response variability and the effect of participant devices on both reaction time and sensitivity. Patients with PD have impaired sensitivity in discriminating between durations of both visual and auditory stimuli compared to healthy controls. Though initially designed as an in-person study, because of the pandemic the experiment was adapted into an online study. This adaptation provided a unique opportunity to enroll a larger number of international participants and use this study to evaluate the feasibility of future virtual studies focused on cognitive impairment. To our knowledge this is the only time perception study, focusing on PD, which measures the differences in perception using both auditory and visual stimuli. The cohort involved is the largest to date, comprising over 800 participants.

## Introduction

Cognitive deficits are common in Parkinson’s disease (PD) and range from mild cognitive impairment to dementia, often dramatically reducing quality of life (Aarsland et al., 2021). Physiological models have shown attention and memory are predicated on the brain’s ability to process time (Pouthas and Perbal, 2004). An impairment in this neural ability (Allman and Meck, 2011) can manifest as mild or severe cognitive impairment (El Haj and Kapogiannis, 2016). Complex neural pathways (Polti et al., 2018) are responsible for us, as individuals, processing time in a manner that is concurrent with one another. The basal ganglia (BG) play an important role (Cope et al., 2014) in this domain.

A hallmark feature of PD (Jankovic and Tan, 2020) is the progressive degeneration of dopaminergic neurons in the substantia nigra (SN) pars compacta of the BG. A loss of dopamine manifests as characteristic motor symptoms such as tremors, fluctuations in gait, and rigidity. The BG is also hypothesized to act as a temporal pacemaker (Bizo and White, 1994) regulated by dopamine to allow us to perceive time. Dopamine alters subjective time perceived in the seconds to minutes range in both animals and humans (Meck, 1996), and dopamine-antagonists’ receptor affinity is known to negatively correlate with perceived duration. The accuracy of time perception is improved by activation of dopaminergic neurons, and impaired by their deactivation (Soares et al., 2016). The systems involved are connected through the frontostriatal loop and play an interactive role in duration discrimination of time intervals. In addition to regulating the speed of the internal clock, dopamine also modulates attentional processing of temporal information (Jones and Jahanshahi, 2011). Studies focusing on patients with diseases that affect dopaminergic pathways such as PD (Malapani et al., 2002), Huntington’s disease (Paulsen et al., 2004), and schizophrenia (Rammsayer, 1990) all show impaired time processing.

In addition to time perception being used a clinical biomarker of neurodegeneration, measuring changes in awareness can help shed light on pathophysiology (Mattay et al., 2002). The circuitry involved in time perception is reliant on a host of neural networks (Lad et al., 2020) involving the BG, hippocampus, parietal and insular cortices, and cerebellum. Degenerative damage to these networks may give rise to specific time perception phenotypes.

The aim of this study was to investigate differences in time perception between patients with PD and healthy controls (HCs). Though initially designed as an in-person study, as a consequence of the outbreak of COVID-19 the experiment was adapted into an online-only, virtual study. This adaptation provided a unique opportunity to enroll a larger number of international participants and use this study to evaluate the feasibility of future virtual studies focused on cognitive impairment.

We searched PubMed, Web of Science, and Google Scholar to identify articles published from inception to November 2021, using the keywords “Parkinson’s disease” and “time perception” or “time processing” or “temporal perception,” with English language restrictions. A previous study (Magalhães et al., 2018), consisting of far smaller participant cohorts, have concentrated on in-person visual perception tasks alone. Another study found movement synchronization to visual but not auditory stimuli was impaired by PD (Wu et al., 2020), suggesting a visuomotor timing deficiency in patients. Since the auditory system is known to have higher resolution in temporal perception (Rammsayer et al., 2015) and engage different circuits in the somatosensory system (Comstock et al., 2018), examining both auditory and visual time perception is crucial to better understand timing impairments in PD.

This study aims to measure differences in time perception, using both auditory and visual stimuli, between patients with PD and HCs. We measure differences in sub-second- and second-time intervals. Sensitivity and error in perception as well as the response time are calculated. Additionally, we investigate intra-individual response variability and the effect of participant devices on both reaction time and sensitivity.

To our knowledge this is the only time perception study, focusing on PD, which measures the differences in perception using both auditory and visual stimuli. The cohort involved is the largest to date, comprising over 800 participants.

The initial intention was to conduct this study at in-person visits. However, COVID-19 related travel restrictions led to its adaptation to an online platform. This had unexpected benefits: firstly, the number of possible participants increased substantially, and secondly, an international cohort of participants was able to be recruited. In addition, conducting the study online in participants’ homes using their personal devices, afforded us the opportunity to investigate the feasibility and accuracy of virtual studies using touch screens and home computers. It is also important to highlight the ability of virtual studies to protect participants and remain relatively robust during a long-lasting pandemic whilst still producing coherent data.

## Materials and methods

The protocol was approved by the University of Oxford Medical Sciences Division Research Ethics Committee (R64730/RE001).

### Participants

Participants were recruited to PD and HC groups. Participants in the PD group had an established diagnosis of PD with an age at disease onset of over 50 years, had no neurological conditions other than PD, no conditions affecting hearing or vision, and had not undergone deep brain stimulation surgery. HC participants were above 50 years of age without any neurological conditions.

### Stimuli and procedure

We tested participants’ time perception using both visual and auditory stimuli. The visual stimulus was a clock figure displayed on a screen, of a design inspired by Benjamin Libet’s seminal time perception experiment (Libet et al., 1983). The design is included in Supplementary material. The auditory stimuli consisted of pure (sinusoidal) tones at 440 Hz generated using MATLAB.

The tasks were completed online using participants’ own devices, which including smartphones, tablet computers, laptops, and desktops. The study was hosted on the Gorilla platform (Anwyl-Irvine et al., 2020)^1^ and all tasks were implemented in JavaScript. All participants first completed a questionnaire collecting information on age, gender, duration of PD, other medical conditions, and current medications.

Within each group (PD or HC), participants were then randomly assigned to one of two subgroups. One subgroup, referred to as the “subseconds” group, were tested using stimuli with a reference duration of 500 ms for both visual and audio tasks. The other subgroup, referred to as the “seconds” group, were tested using stimuli with a reference duration of 1500 ms for both visual and audio tasks. For each participant, the order of the two tasks (visual then audio or vice versa) was chosen randomly. The auditory task was preceded by a test audio clip and instruction to participants to adjust their devices’ audio levels so that they could hear the audio clip clearly.

Both visual and audio tasks started with two practice trials to familiarize the participant with the task and their response method (keyboard or touch screen response), followed by up to 100 actual trials. Each trial began with a blank screen for 750 ms, followed by a standard stimulus (auditory or visual) of fixed duration (500 ms for the subseconds group and 1,500 ms for the seconds group), then another blank screen for 750 ms, followed by a comparison stimulus of the same modality, with a variable duration that could be longer or shorter than the standard stimulus. Participants were asked to respond by indicating whether the comparison stimulus duration was longer or shorter than the standard stimulus duration.

There were 100 possible comparison stimulus durations ranging from 0.5 to 1.5 times the standard stimulus duration with increments of 1% of the first stimulus duration, and the standard duration was not re-used (i.e., for the subseconds group the comparison stimulus durations ranged from 250 to 750 ms with 5 ms increments excluding 500 ms, and for the seconds group the comparison stimulus durations ranged from 750 to 2,250 ms with 15 ms increments excluding 1,500 ms). Comparison stimulus durations were sampled randomly without replacement from the pool of possible values.

We performed at least 55 trials in each participant; this choice was based upon visual inspection of pilot datasets, in which this was the minimum number required for stability of the estimated parameters. Trials were continued beyond this until the rolling 10-point coefficient of variation of estimates of the logistic regression slope was less than 2% for 5 consecutive trials (see Supplementary material for pseudocode). Meeting both requirements meant that additional responses were not likely to significantly change the slope. A limit of 100 trials was imposed to mitigate possible contamination of the data by fatigue effects.

### Sample size

We calculated the minimum sample size needed for the study using the G*Power software (Faul et al., 2007), selecting a medium effect size of 0.40 with 90% power at a significance level of *p* < 0.05, assuming normally distributed populations with equal variance. A total of 113 subjects for each group were determined to be necessary for analysis using a Wilcoxon–Mann–Whitney test. As participants were randomized to complete tests with one of two different time intervals, a total of 226 participants were required for each of the PD and HC groups. Recruitment proved to be straightforward and this minimum was easily exceeded.

### Data analysis

**FIGURE 1:**
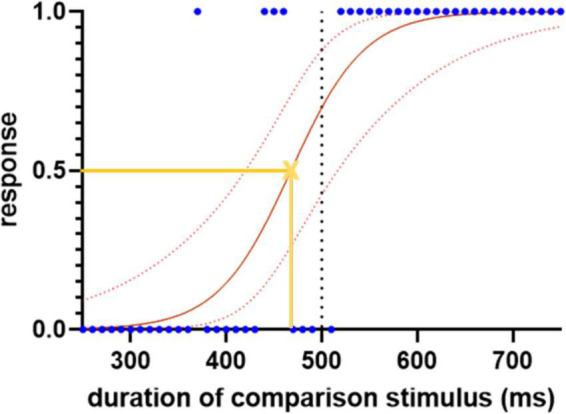
Sample logistic regression curve for the subseconds interval, demonstrating the spread of the responses, 0 indicates “shorter” and 1 indicates “longer.” The blue dots represent each response. The solid red line represents the fitted logistic regression curse and the dotted red lines indicate the 95% confidence intervals. The yellow cross represents the point of subjective equality where the participant has equal likelihood to choose either “shorter” or “longer” responses. The slope of the curve at this point, and the shift of its 50% point from the standard duration (500 ms), are used to quantify the sensitivity and error of time perception, respectively.

For each trial, a response of “shorter” was assigned the value 0 and “longer” the value 1. The responses were then plotted against the duration of the comparison stimulus and fitted with a logistic regression curve (Figure 1). The slope of the curve at its 50% point is a measure of the sensitivity of time perception (a higher value implying a sharper cut-off between “shorter” and “longer”). The left or right shift of its 50% point from the standard duration quantifies any systematic error of comparison of the duration of first (standard) and second (comparison) stimuli. It is calculated as the proportional shift away from the standard duration.

The model equation was:


(1) $$P=\frac{1}{1+e^{-\left(\beta_{0}+\beta_{1}*X\right)}}$$

where *P* is the probability of giving a response of “longer,” *X* is the duration of the comparison stimulus, *e* is the base of the natural logarithm, β_0_ is the intercept, and β_1_ is an inverse scale parameter, with higher values of β_1_ indicating a steeper slope and thus more sensitive temporal discrimination. The point at which participants choose between the two responses with equal probability is −β_0_/β_1_. From the two coefficients of the logistic regression model β_0_ and β_1_, sensitivity and error of time perception are calculated as:


(2) $$S\,e\,n\,s\,i\,t\,i\,v\,i\,t\,y=\ln\beta_{1}$$

(3) $$E\,r\,r\,o\,r=\left(-\frac{\beta_{0}}{\beta_{1}}-s\,t\,a\,n\,d\,a\,r\,d\right)/s\,t\,a\,n\,d\,a\,r\,d$$

where standard is the duration of the comparison stimuli in ms.

Because the distribution of logistic regression slopes was highly positively skewed in both PD and control groups, a logarithmic transformation was performed to permit parametric analysis.

To ensure participants understood the task, and in order to only include trials where an active discrimination was made, tasks with negative slopes were excluded as well as participants with invalid or missing audio or visual task data. In line with established procedures in reaction time research (Hultsch et al., 2002), all trials with response times shorter than 250 ms or longer than three standard deviations above the group mean (PD, HC) were removed from analysis. Response time is defined as the time between the end of last stimulus to the detected response from the participant in milliseconds.

Intraindividual variability (IIV) of reaction time has been speculated as a cognitive predictor of mild cognitive disorders (Bielak et al., 2010; Cherbuin et al., 2010). Intraindividual standard deviation is one of the simplest indices that can be computed to quantify IIV, however, this does not take into account the systematic time-related effects or the different comparison durations on the response time. To factor out the effects associated with comparison duration, residual scores from linear regression of response time against comparison duration were taken and converted to *T* scores by trials before the standard deviation of the *T* scores were taken as IIV uncontaminated by systematic effects and confounding factors.

Bonferroni correction was used to correct for multiple comparisons using the Mann–Whitney test.

## Results

### Demographics

A total of 320 patients with PD were recruited through a multitude of Parkinson’s associations worldwide and 620 HCs above the age of 50 were recruited using Prolific (Table 1).^2^ A total of 15 patients and 51 controls were excluded from analysis due to not understanding the task, not completing both auditory and visual tasks, or health conditions. Of the 292 patients and 569 controls included for analysis, the mean age in the two groups was similar (66.5 years for PD vs. 65.8 years for HC). The groups did not differ significantly in gender (54% female in the PD group vs. 60% female in HC; *p* = 0.10, 2 sample proportion *z*-test). Within the PD group, the average disease duration was 6.5 years, with participants ranging from newly diagnosed to up to 28 years. Most PD patients were taking levodopa (86%) while some were taking alternative PD medications (10%) or no medication at all (3%). Self-reported time since last medication ranged from 0 to 24 h with a median of 2.5 h. The majority of participants used a computer as opposed to a phone or tablet to complete the task (77% in PD and 84% in HC). Windows was the most common operating system used in this cohort of participants (58%), followed by macOS (19%).

**TABLE 1:** Demographics table of Parkinson’s disease (PD) group and healthy control (HC) group performing the seconds or subseconds task.

	Subseconds		Seconds		Overall	
	HC (*N* = 269)	PD (*N* = 129)	HC (*N* = 300)	PD (*N* = 163)	HC (*N* = 569)	PD (*N* = 292)
**Age (years)**						
Mean (SD)	65.6 (5.35)	66.5 (6.83)	66.1 (5.54)	66.5 (7.52)	65.8 (5.45)	66.5 (7.21)
Median (Min, Max)	65.0 (50.0, 87.0)	66.0 (50.0, 85.0)	65.5 (52.0, 86.0)	67.0 (50.0, 85.0)	65.0 (50.0, 87.0)	67.0 (50.0, 85.0)
**Sex**						
Male	100 (37.2%)	61 (47.3%)	127 (42.3%)	73 (44.8%)	227 (39.9%)	134 (45.9%)
Female	169 (62.8%)	68 (52.7%)	173 (57.7%)	90 (55.2%)	342 (60.1%)	158 (54.1%)
**Handedness**						
Left	35 (13.0%)	13 (10.1%)	29 (9.7%)	19 (11.7%)	64 (11.2%)	32 (11.0%)
Right	234 (87.0%)	111 (86.0%)	269 (89.7%)	140 (85.9%)	503 (88.4%)	251 (86.0%)
Ambidextrous	0 (0%)	5 (3.9%)	2 (0.7%)	4 (2.5%)	2 (0.4%)	9 (3.1%)
**Ethnicity**						
Asian	2 (0.7%)	1 (0.8%)	4 (1.3%)	1 (0.6%)	6 (1.1%)	2 (0.7%)
Hispanic or Latino	2 (0.7%)	0 (0%)	1 (0.3%)	2 (1.2%)	3 (0.5%)	2 (0.7%)
Other (please specify)	5 (1.9%)	1 (0.8%)	5 (1.7%)	1 (0.6%)	10 (1.8%)	2 (0.7%)
White	250 (92.9%)	102 (79.1%)	284 (94.7%)	157 (96.3%)	534 (93.8%)	259 (88.7%)
Black or African American	0 (0%)	0 (0%)	3 (1.0%)	0 (0%)	3 (0.5%)	0 (0%)
Jewish	0 (0%)	0 (0%)	1 (0.3%)	0 (0%)	1 (0.2%)	0 (0%)
**PD duration (years)**						
Mean (SD)	NA (NA)	6.35 (4.88)	NA (NA)	6.57 (4.65)	NA (NA)	6.47 (4.74)
Median (Min, Max)	NA (NA, NA)	5.00 (0.0137, 24.0)	NA (NA, NA)	5.00 (0.00800, 28.0)	NA (NA, NA)	5.00 (0.00800, 28.0)
**PD medication**						
*De novo*	0 (0%)	4 (3.1%)	0 (0%)	6 (3.7%)	0 (0%)	10 (3.4%)
Levodopa	0 (0%)	110 (85.3%)	0 (0%)	142 (87.1%)	0 (0%)	252 (86.3%)
Others	0 (0%)	14 (10.9%)	0 (0%)	15 (9.2%)	0 (0%)	29 (9.9%)
**Time since last med (hours)**						
Mean (SD)	NA (NA)	3.21 (3.49)	NA (NA)	3.33 (3.37)	NA (NA)	3.28 (3.41)
Median (Min, Max)	NA (NA, NA)	2.00 (0, 21.0)	NA (NA, NA)	3.00 (0, 24.0)	NA (NA, NA)	2.50 (0, 24.0)
**Device**						
Computer	221 (82.2%)	102 (79.1%)	255 (85.0%)	122 (74.8%)	476 (83.7%)	224 (76.7%)
Mobile	33 (12.3%)	12 (9.3%)	28 (9.3%)	15 (9.2%)	61 (10.7%)	27 (9.2%)
Tablet	15 (5.6%)	15 (11.6%)	17 (5.7%)	26 (16.0%)	32 (5.6%)	41 (14.0%)
**Operating system**						
Android	28 (10.4%)	11 (8.5%)	27 (9.0%)	17 (10.4%)	55 (9.7%)	28 (9.6%)
Chromium	7 (2.6%)	1 (0.8%)	8 (2.7%)	1 (0.6%)	15 (2.6%)	2 (0.7%)
iOS	23 (8.6%)	16 (12.4%)	22 (7.3%)	24 (14.7%)	45 (7.9%)	40 (13.7%)
Linux	1 (0.4%)	1 (0.8%)	1 (0.3%)	1 (0.6%)	2 (0.4%)	2 (0.7%)
Mac	32 (11.9%)	37 (28.7%)	52 (17.3%)	41 (25.2%)	84 (14.8%)	78 (26.7%)
Ubuntu	5 (1.9%)	1 (0.8%)	1 (0.3%)	0 (0%)	6 (1.1%)	1 (0.3%)
Windows	173 (64.3%)	62 (48.1%)	189 (63.0%)	79 (48.5%)	362 (63.6%)	141 (48.3%)

### Duration discrimination of Parkinson’s disease vs. healthy controls

**FIGURE 2:**
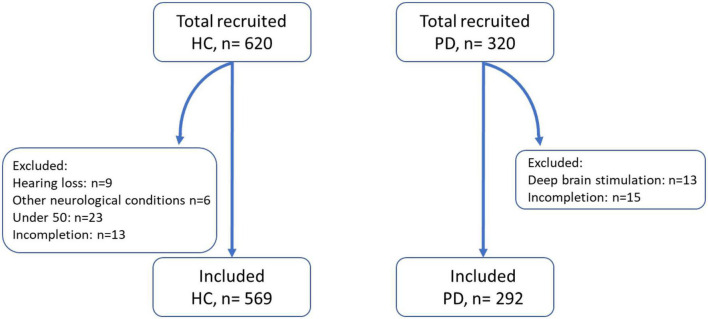
Number of control and patient participants recruited, excluded, and included for analysis. HC, healthy control; PD, Parkinson’s disease patients. Other neurological conditions included multiple sclerosis and epilepsy.

**FIGURE 3:**
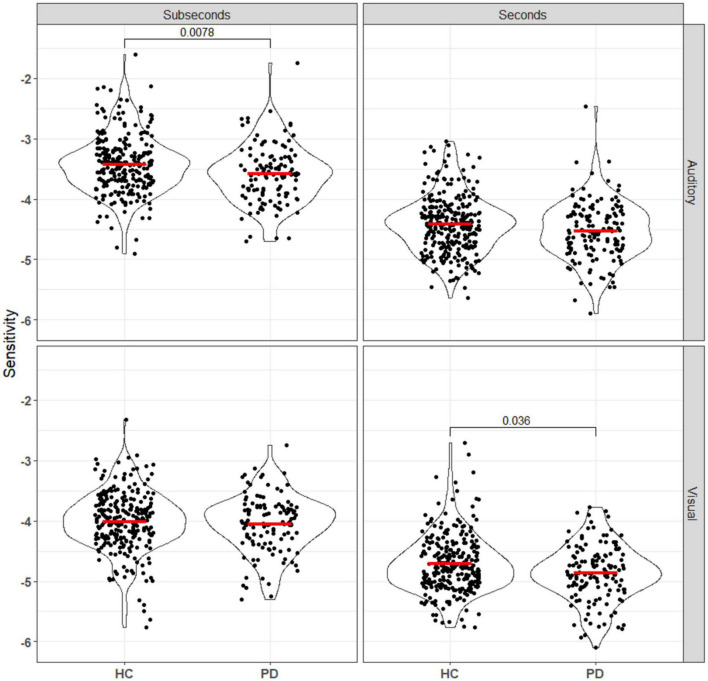
Sensitivity, calculated using equation (Pouthas and Perbal, 2004), of all patients and controls to both auditory and visual stimuli in the seconds and subseconds intervals. PD patients showed impaired time perception to auditory stimuli in the subseconds range and visual stimuli in the seconds range. Annotations denote adjusted *p*-values after Bonferroni correction, only statistically significant *p*-values are shown.

Duration discrimination sensitivity was represented by log values of the slope of each logistic regression curve, where a larger value represents steeper slope and better sensitivity. Participants were randomized to either the seconds or subseconds group and two logistic regression models were fitted for each participant, one for each of the audio and the visual task. Mann–Whitney U tests were used to compare the sensitivity and error between the PD and HC groups for each combination of reference duration (seconds vs. subseconds) and stimulus modality (auditory vs. visual) (Figure 2). After Bonferroni correction, statistical significance (*p*_*unc*_ < 0.0125) was observed in visual task in the seconds range and auditory task in the subseconds range. In the seconds interval, patients had a significantly worse visual sensitivity (auditory: −4.42 ± 0.47; visual: −4.71 ± 0.47) compared to that of controls (auditory: −4.55 ± 0.49; visual: −4.89 ± 0.46). In the subseconds interval, patients had a significantly worse auditory sensitivity (−3.43 ± 0.50 HC vs. −3.60 ± 0.48 PD) compared to that of controls. PD patients consistently overestimate duration of the second auditory stimulus compared to the first in both seconds (*p* = 0.0065) and subseconds (*p* < 0.001) interval compared to the control group (Figure 3). This shift is absent in both visual tasks.

### Intra-individual variability

Standard deviations of response times and IIV for each auditory and visual stimuli in the seconds and subseconds range are summarized in Table 2. There is no significant difference in response time variability between patient and control groups after Bonferroni correction.

**TABLE 2:** Standard deviation (SD) and intra-individual variability (IIV) of response time in HC and PD groups responding to auditory and visual stimuli in the seconds and subseconds range.

	Subseconds			Seconds		
	HC (*N* = 256)	PD (*N* = 120)	*p*-value	HC (*N* = 275)	PD (*N* = 144)	*p*-value
**Auditory response time SD**						
Mean (SD)	447 (310)	570 (506)	0.014	458 (327)	519 (283)	0.049
Median (Min, Max)	350 (105, 2,740)	405 (115, 4,100)		364 (106, 2,710)	448 (148, 1,470)	
**Visual response time SD**						
Mean (SD)	445 (324)	631 (918)	0.033	477 (390)	749 (1,760)	0.07
Median (Min, Max)	368 (93.3, 2,710)	440 (85.2, 9,390)		380 (113, 3,330)	508 (133, 20,600)	
**Auditory response time IIV**						
Mean (SD)	8.54 (0.796)	8.73 (0.761)	0.03	8.56 (0.834)	8.65 (0.858)	0.288
Median (Min, Max)	8.60 (6.16, 9.95)	8.66 (7.21, 9.95)		8.60 (6.93, 10.0)	8.66 (6.71, 10.0)	
**Visual response time IIV**						
Mean (SD)	8.89 (0.762)	9.06 (0.775)	0.055	8.67 (0.787)	8.76 (0.814)	0.259
Median (Min, Max)	9.00 (7.14, 9.95)	9.14 (7.14, 9.95)		8.72 (6.86, 10.0)	8.77 (6.71, 10.0)	

*p*-Values are from unpaired *t*-tests. There is no statistically significant difference in the variability in response time after Bonferroni correction.

## Discussion

This study has shown that patients with PD have impaired sensitivity in discriminating between durations of both visual and auditory stimuli compared to HCs. This effect has not been found in previous studies (Terao et al., 2021); likely due to the greatly increased sample sizes and thus statistical power permitted by the online approach taken here.

### Limitations

To the authors’ knowledge, this is the first study of time perception in PD to have been conducted entirely online. While this has enabled us to greatly increase sample sizes, several sources of variability are also introduced by this method of delivery.

Firstly, computer hardware and software varied significantly between participants (see Supplementary Data). This could have affected recording of reaction times, but also stimulus delivery, e.g., by slowdown due to multiple applications being open simultaneously, by variable screen refresh rates, or by the use of wireless audio peripherals. Gorilla Experiment Builder, which was used to deliver the experiment, is designed primarily to mitigate these inconsistencies between systems, but cannot eliminate them entirely (Anwyl-Irvine et al., 2020).

Secondly, we relied entirely on self-report measures; participants were not assessed by a clinician as part of the study, nor were any data from their medical records available. Patients on antiparkinsonian medications were asked to list their medications and estimate the number of hours since last taking medication. Thus, patients were not in a well-defined “on” or “off” state during testing, and levodopa equivalent dose could not be estimated from this information. While a previous small study found no effect of levodopa on PD patients’ ability to discriminate between durations around 50 ms (Guehl et al., 2008), the vastly shorter stimulus duration in that study may have probed intracortical rather than corticostriatal processing, so the effect of this on our experiment is unknown.

Finally, the experimental environment cannot be controlled as tightly as is typical for in-person experiments. Factors intrinsic to the task such as stimulus luminance/loudness and contrast, which are known to affect duration perception for visual stimuli (Terao et al., 2008), would have been under control of the participant’s device. Similarly, extrinsic factors (i.e., distractors) would have varied widely between participants. In a hypothetical extreme case, for example, a participant might have completed the task on a bus. This means they would have been exposed to a wide range of simultaneous auditory and visual stimuli in an environment with rapidly changing background luminance and noise, all while attending another time perception task at a different timescale (estimating the interval of time elapsed since getting on or passing a previous stop in order to leave the bus at the right time).

Despite these limitations, online delivery of this study has permitted group sizes an order of magnitude greater than those used in previous studies, and recruitment across a far larger geographical area, leading to greater statistical power.

### Toward an integrative model of Parkinsonian deficits in suprasecond time processing

There is a significant body of evidence to support the theory that time perception is mediated by different mechanisms at different timescales (Buhusi and Meck, 2005; Rammsayer and Ulrich, 2012), and the BG are particularly implicated in the processing of longer (suprasecond) durations. Per the striatal beat-frequency model (Matell and Meck, 2000), cortico-striato-thalamo-cortical (CSTC) loop circuits facilitate the comparison of cortical oscillations at the start and end of an interval. Based on functional neuroimaging evidence, a role for dopaminergic neurons in the *substantia nigra pars compacta* (SNc) in this process as a “perceptual starting gun” for interval timing by resetting the phase of these oscillations has been proposed (Jahanshahi et al., 2006), which has since been backed up by a study in mice (Soares et al., 2016).

Taken together, this model would predict that PD, which is characterized by degeneration of SNc dopaminergic neurons and widespread striatal pathology, produces an impairment in suprasecond interval perception across modalities. However, the nature of this impairment is complex, as one must consider the interacting effects of a hypodopaminergic state, the resulting chronic dysfunction (hyperexcitability) in striatal dopamine-*recipient* neurons, and any dopaminergic medication effects (Liang et al., 2008; Singh et al., 2014). Thus, we expected to see both a loss of sensitivity on average, and an increase in inter-individual variability within the PD population. Additionally, distortions in unimodal perception at subsecond timescales can be sufficient to give rise to modality-specific effects at suprasecond timescales in hierarchical models where suprasecond timekeeping is amodal (Stauffer et al., 2012; Rammsayer et al., 2015); in light of this, it was surprising that the PD group had significant decreases in auditory sensitivity in only the subsecond range in our data (Figure 3). Given the size of the difference in sample medians, it is likely that future online-only study designs would need even larger group sizes to detect subtle effects like this.

### Perceptual bias across timescales

**FIGURE 4:**
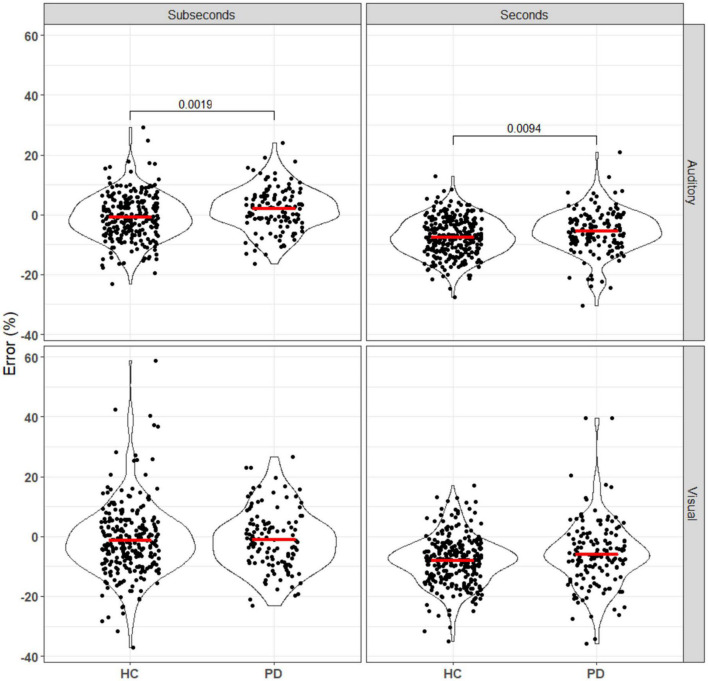
Error of patients and controls in comparing auditory and visual stimuli in the seconds and subseconds intervals (Equation 3). No significant difference was seen between the patient and control groups for the visual stimuli. For the auditory stimuli, PD patients overestimated the standard duration compared to the controls. Annotations denote adjusted *p*-values after Bonferroni correction, only statistically significant *p*-values are shown.

Biases in temporal perception have also been observed in this study, with systematic differences in the relative perceived durations of the standard (first) and comparison (second) stimuli (Figure 4). As the stimuli were not perceived simultaneously, several processes can affect this through changes in the perception of the first duration, the memory of the first duration, and the perception of the second duration.

The effect first observed by Terao et al. (2021), in which subjective error shifted from more positive to more negative as stimulus length increased for both groups, was replicated here (all comparisons *p*0.001 after Bonferroni correction; see Supplementary Data). However, the observed difference between the control and PD groups was in the opposite direction, with our data suggesting a more positive error in PD patients.

Care must be taken when interpreting these effects, as perception may change based on previous stimuli or peri-stimulus sensory or motor events. For example, repetition of a stimulus shortens its perceived duration (Eagleman, 2008; Pariyadath and Eagleman, 2012; Cai et al., 2015), and saccades made around the onset of a visual stimulus also compress its subjective duration (Morrone et al., 2005; Terao et al., 2008). As the external sensory environment of our participants was unknown, it is difficult to estimate the degree to which these effects are present in our data.

Additionally, fatigue may introduce biases which may differ both between experimental groups and within the patient population. On balance, it is difficult to completely adjust for these in studies such as ours, but further studies should attempt to quantify these effects specifically, with a larger number of participants and possibly a fixed, higher number of trials to elicit fatigue in HCs.

### Developing cognitive biomarkers for Parkinson’s disease

Aberrant time perception in PD could provide some insight into disease progression and severity. Perceptual error in auditory duration comparison correlated with mini-mental state examination (MMSE) score in a recent study (Terao et al., 2021), and should be studied longitudinally in a prospective cohort study. Additionally, these deficits may exhibit consistent treatment effects: two studies have shown improvements of deep brain stimulation of the subthalamic nucleus (STN DBS) on time perception in auditory interval discrimination at subsecond time scales, and motor time production at suprasecond timescales (Koch et al., 2004; Guehl et al., 2008). These effects could potentially be exploited for treatment optimization, but should be studied in greater detail as there is a degree of task dependence. While motor and sensory midpoints in an interval bisection task are indistinguishable (Levy et al., 2015), the relationship between bias and stimulus length may be reversed between motor and sensory tasks (Terao et al., 2021).

Data on disease duration and medication were collected as part of our study, and will be included in a future analysis.

## Conclusion

Our findings demonstrate modality- and stimulus length-specific differences in duration perception in patients with PD vs. HCs. These differences may shed light on timekeeping systems and the pathophysiology of PD, but require further study and careful examination of the effects observed as they can be sensitive to task design and experimental conditions.

## Data availability statement

The original contributions presented in this study are included in the article/Supplementary material, further inquiries can be directed to the corresponding author.

## Ethics statement

The protocol was approved by the University of Oxford Medical Sciences Division Research Ethics Committee (R64730/RE001). The patients/participants provided their written informed consent to participate in this study.

## Author contributions

ZS, SP, CA, and JF designed and oversaw the study. ZS and CA oversaw the testing and implementation of the interactive website (*via* Gorilla). All authors critically reviewed various versions of the manuscript. JF and CA oversaw the statistical analysis for the study. All authors have seen and have access to the whole dataset presented here.

## Footnotes

1
www.gorilla.sc

2
www.prolific.co
