# Supplementary material for: Parkinson’s disease deficits in time perception to auditory as well as visual stimuli – A large online study
Source: Front Neurosci. 2022 Oct 20;16:995438. doi: 10.3389/fnins.2022.995438 (PMC9632441; doi:10.3389/fnins.2022.995438)
Supplement: Supplementary file 1 [file Data_Sheet_1.docx]

**Supplementary Materials**

### Study Design


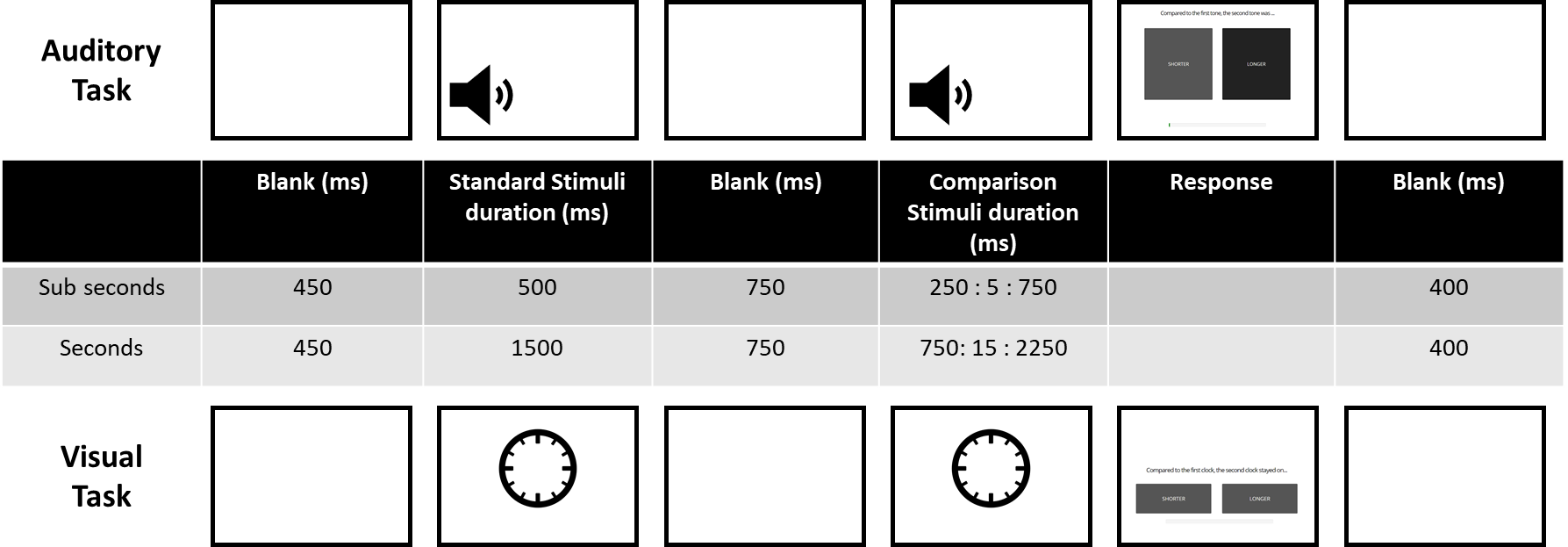


##
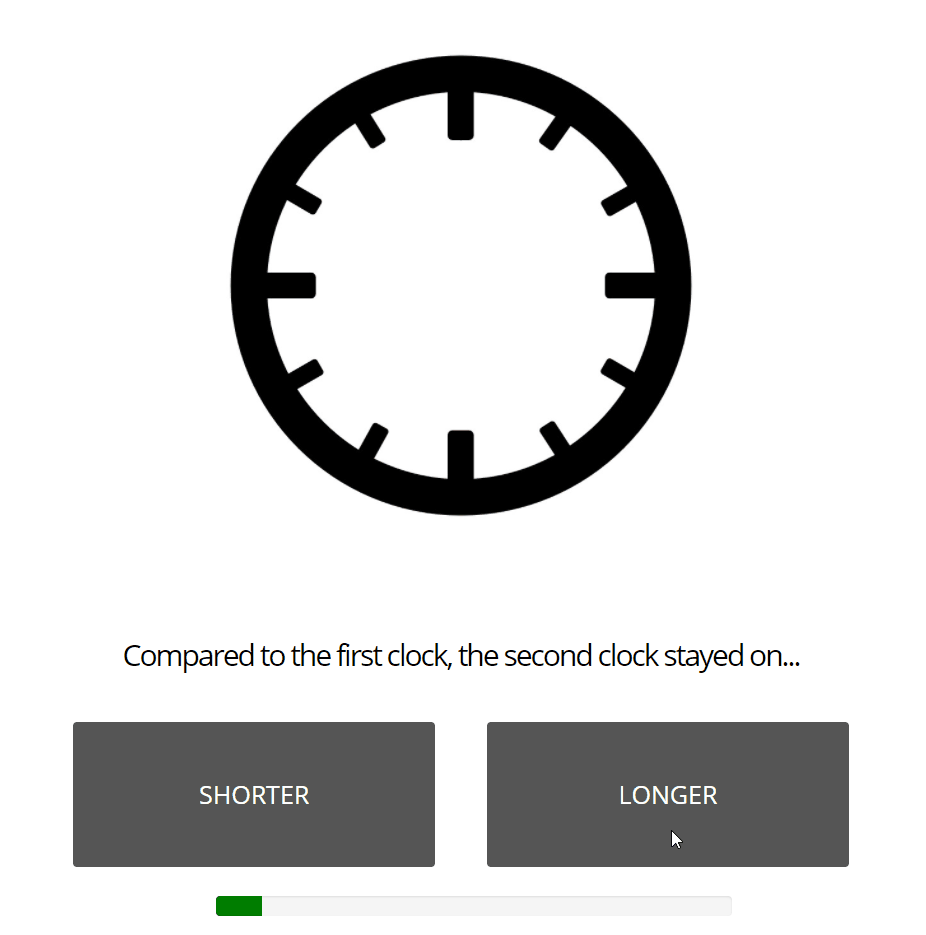

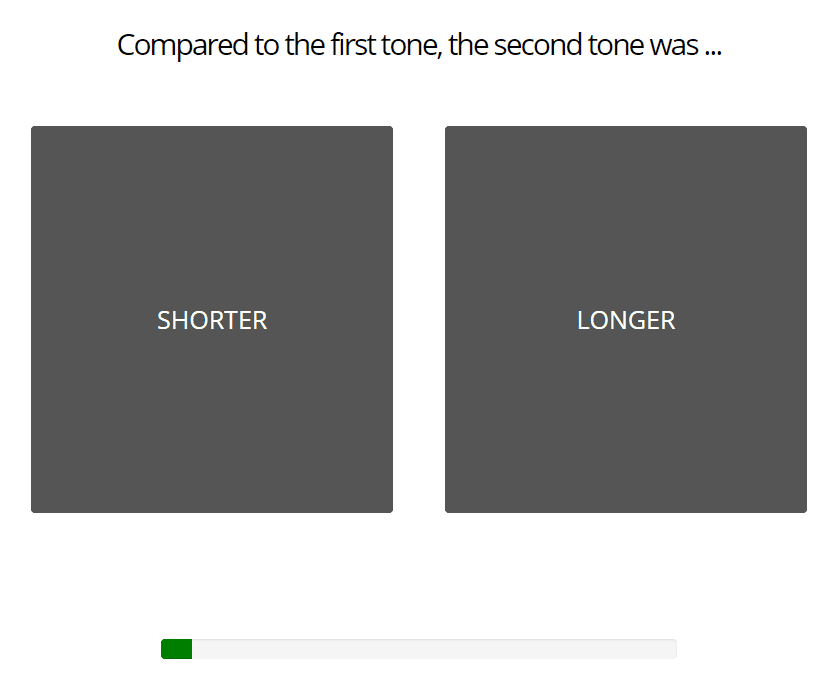

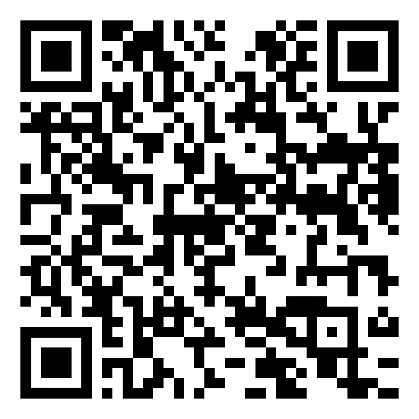


## Pseudocode for early stopping algorithm

**set** durations $\leftarrow$ [standard_duration $\times$ [0.50 **to** 1.50 **by** 0.01 **except** 1.00]],

trial_durations $\leftarrow$ random_permutation(durations),

responses $\leftarrow$ [],

$\beta_{0} \leftarrow$ [], $\beta_{1} \leftarrow$ [],

cv $\leftarrow$ [**repeat** NA **for** [1 **to** 50]],

i_trial $\leftarrow$ 1,

**while** i_trial $\leq$ 100 **do**

play_stimulus(trial_durations_i_trial_)

**set** responses_i_trial_ $\leftarrow$ **input**

**if** i_trial > 40 **do**

**set** ($\beta_{0,}$_i_trial_,$\beta_{1,}$_i_trial_) $\leftarrow$ fit_logistic_regression(trial_durations_[1_ **_to_** _i_trial]_, responses)

**end if**

**if** i_trial > 50 **do**

**set** cv_i_trial_ $\leftarrow$ stddev($\beta_{1,}$_[i_trial-9_ **_to_** _i_trial]_)/mean($\beta_{1,}$_[i_trial-9_ **_to_** _i_trial]_)

**end if**

**if** i_trial > 55 **and** all(cv_[i_trial-4_ **_to_** _i_trial]_ < 0.02) **then exit**

**set** i_trial $\leftarrow$ i_trial + 1

**end while**

### Within Group Errors (paired Wilcoxon with Bonferroni)

***
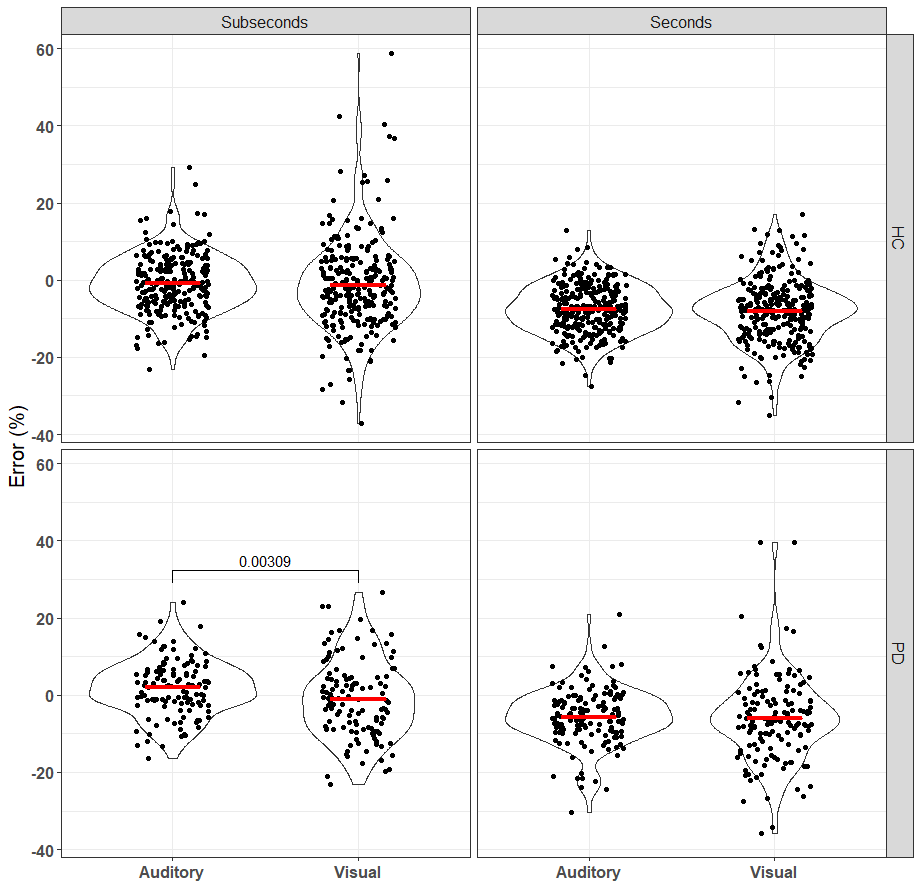
***

### Between Group Errors (Mann-Whitney with Bonferroni)


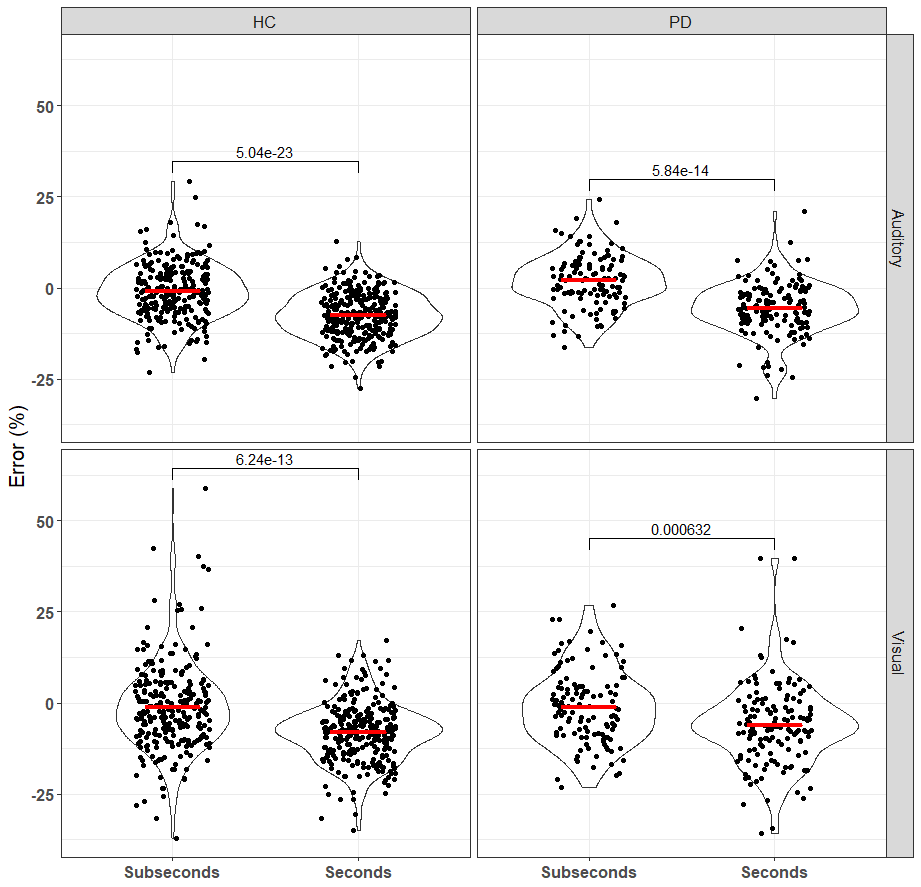


### Device


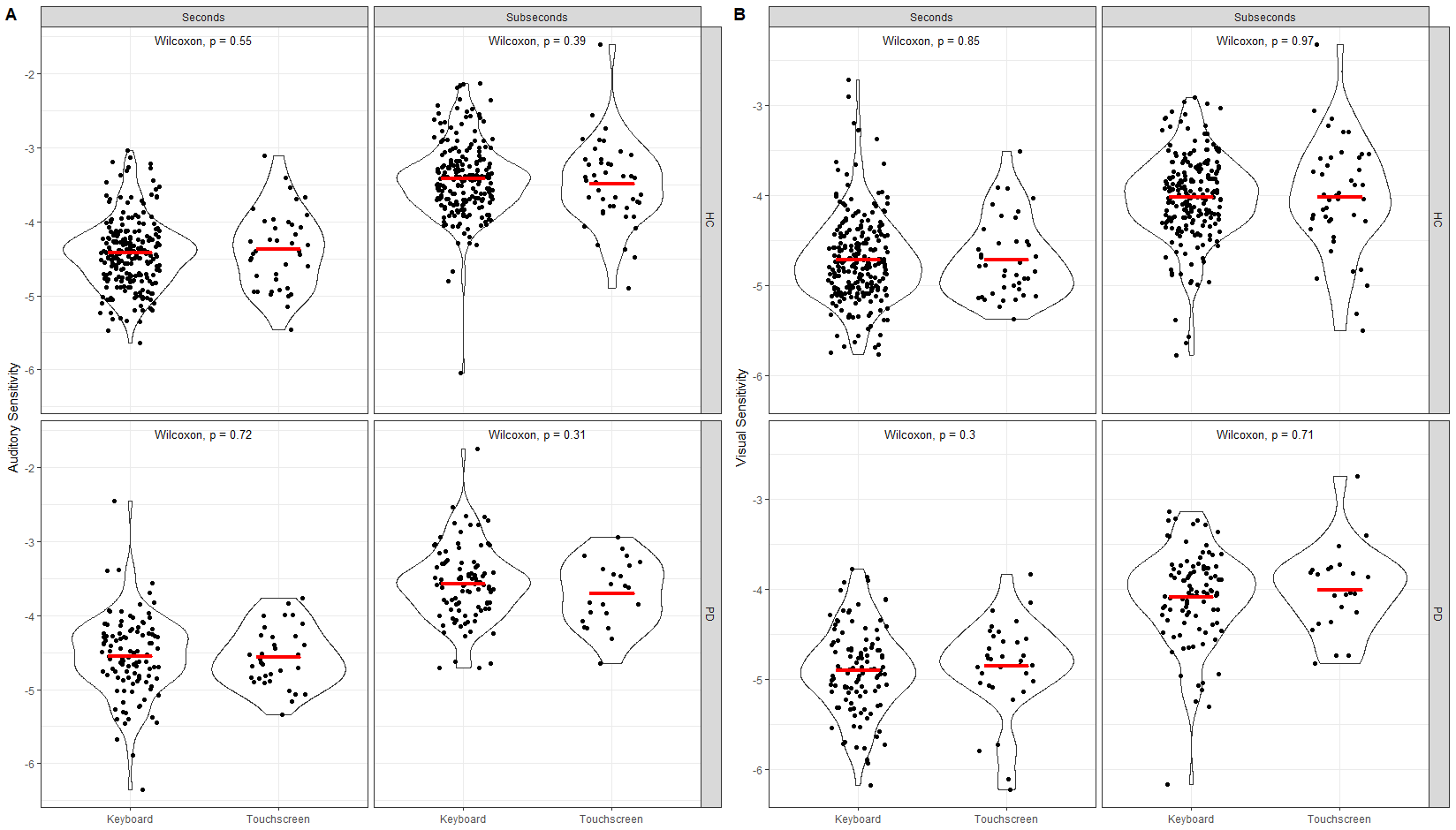


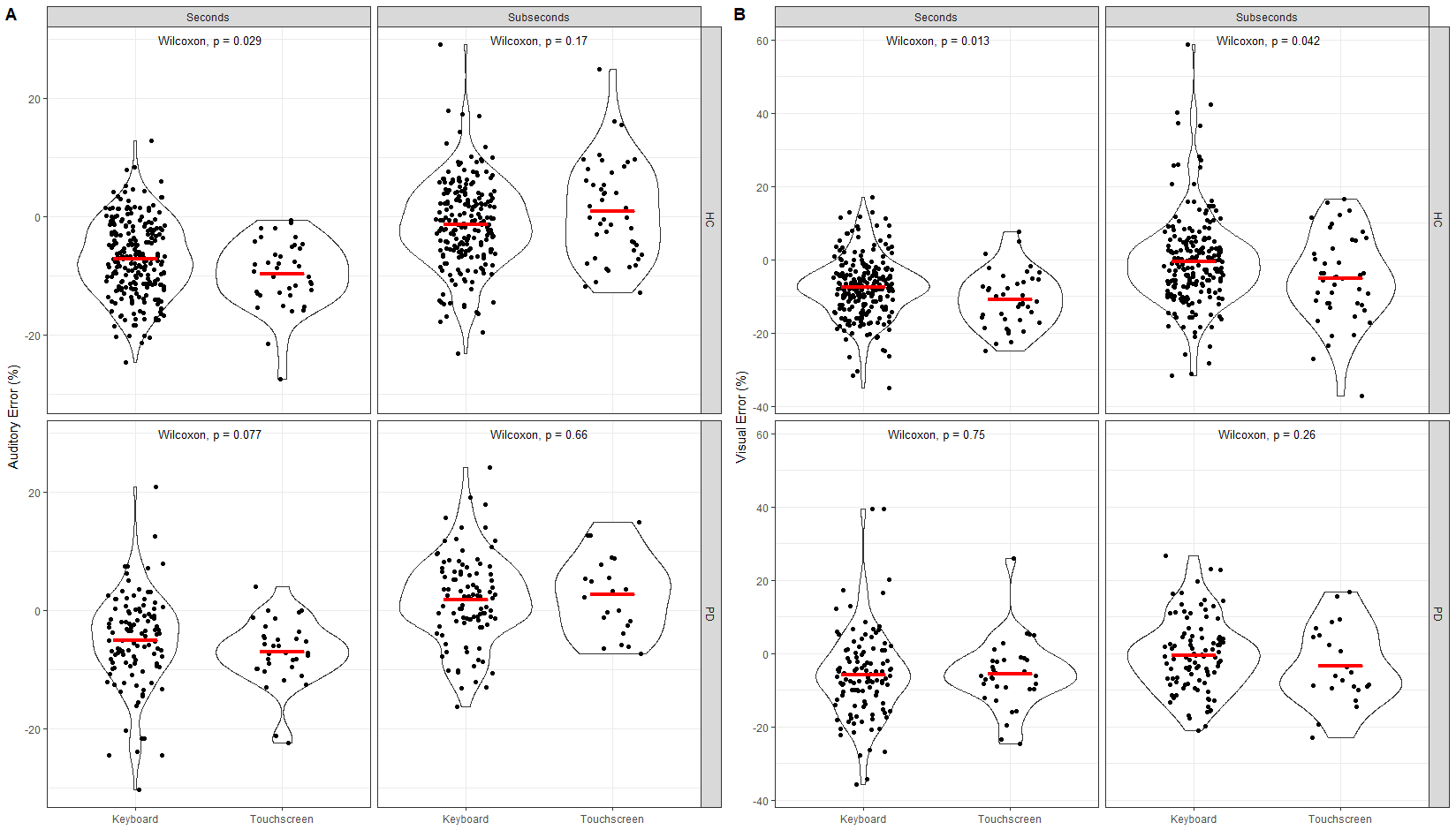


### Intra-Individual Variability


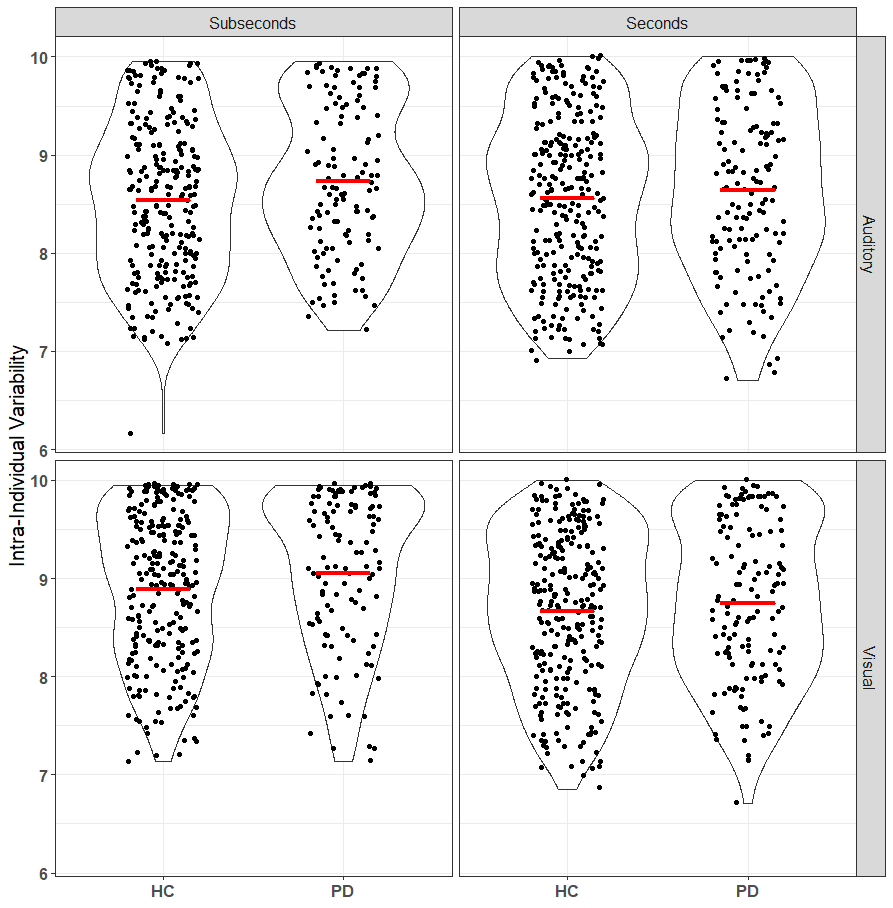


Intraindividual response time variability (SD of response times per individual per task)
